# Dropouts and Compliance in Exercise Interventions Targeting Bone Mineral Density in Adults: A Meta-Analysis of Randomized Controlled Trials

**DOI:** 10.1155/2013/250423

**Published:** 2013-06-03

**Authors:** George A. Kelley, Kristi S. Kelley

**Affiliations:** Meta-Analytic Research Group, Robert C. Byrd Health Sciences Center, Department of Biostatistics, School of Public Health, West Virginia University, Morgantown, WV 26506-9190, USA

## Abstract

*Background.* Dropouts and compliance to exercise interventions targeting bone mineral density (BMD) in adults are not well established. The purpose of this study was to address that gap. *Methods.* Meta-analysis of randomized controlled exercise intervention trials in adults ≥18 years of age. The primary outcomes were dropouts in the exercise and control groups as well as compliance to the exercise interventions. A random-effects model was used to pool results. Moderator analyses were conducted using mixed-effects ANOVA-like models and metaregression. Statistical significance was set at *P* ≤ 0.05. *Results*. Thirty-six studies representing 3,297 participants (1,855 exercise, 1,442 control) were included. Dropout rates in the exercise and control groups averaged 20.9% (95% CI 16.7%–25.9%) and 15.9% (11.8%–21.1%) while compliance to exercise was 76.3% (71.7%–80.3%). For both exercise and control groups, greater dropout rates were associated with studies conducted in the USA versus other countries, females versus males, premenopausal versus postmenopausal women, younger versus older participants, longer studies (controls only), and high- versus moderate-intensity training (exercisers only). Greater compliance to exercise was associated with being female, home- or facility-based exercise versus both, and shorter studies. *Conclusion.* These findings provide important information for researchers and practitioners with respect to exercise programs targeting BMD in adults.

## 1. Introduction

Osteoporosis and the fractures that result from osteoporosis are a major public health problem worldwide. For example, it has been estimated that osteoporosis causes more than 8.9 million fractures annually, resulting in an osteoporotic fracture every 3 seconds [1]. In the United States (USA), the prevalence of osteoporosis and low bone mass includes almost 44 million women and men 50 years of age and older [2]. This represents 55% of US adults aged 50 and older [2]. By the year 2020, it is estimated that more than 61 million women and men in the USA will have osteoporosis or low bone mass [2].

Exercise is a nonpharmacologic intervention that has been recommended for increasing and/or maintaining bone mineral density (BMD) in adults [3, 4]. However, the investigative team is not aware of any previous meta-analytic research that has focused on dropouts and compliance with respect to participants enrolled in nonbehaviorally focused randomized controlled exercise intervention programs targeting BMD in adults. This has important implications from both a research and practice perspective. From a research perspective, knowledge of these potential factors can assist trialists in developing experimental designs that minimize dropout and maximize compliance, thereby allowing one to better identify the true effects of exercise on BMD and other outcomes of interest. From a practice-based standpoint, such knowledge can aid one in determining the feasibility of exercise as a nonpharmacologic intervention for improving BMD in adults. In addition, a recent systematic review that attempted to address the determinants of exercise and physical activity participation in older adults recommended that additional research on this topic was needed [5]. Given the importance of this issue, the purpose of the current study was to use the aggregate data meta-analytic approach to examine dropouts and compliance in exercise interventions targeting BMD in adults, including selected factors associated with such.

## 2. Methods

### 2.1. Study Eligibility Criteria

The current meta-analysis was derived from a large exercise and bone database. Studies were included if they met the following criteria: (1) nonbehaviorally focused randomized trials with a comparative control group (e.g., nonintervention), (2) adults ≥ 18 years of age, (3) participants not engaged in a regular exercise program prior to study enrollment, (4) ground and/or joint reaction force exercise ≥ 24 weeks in which FN and/or LS BMD was assessed, (5) published and unpublished (master's theses and dissertations) studies in any language since January 1989, and (6) data available for dropouts and/or compliance to the exercise intervention. Any studies not meeting all six criteria were excluded. 

 Exercise was defined as that which is planned, structured, and repetitive [4]. Studies were limited to randomized controlled trials in which the exercise intervention lasted at least 24 weeks because most BMD intervention studies last at least this long due to the fact that the bone remodeling process typically takes approximately 24 weeks to complete [6, 7]. Studies were limited to those that assessed FN and/or LS BMD because these are the sites most often studied given that they are the most common sites for fracture [1]. We limited studies to those that assessed BMD because it is considered to be the best predictor for osteoporosis [8]. The year 1989 was chosen as the start date for inclusion since it appeared to be the first time that a randomized controlled trial on exercise and BMD in adult humans was conducted [9].

### 2.2. Data Sources

Potentially eligible studies were derived from a large database that was the result of searching six electronic reference databases (PubMed, Embase, SportDiscus, Cochrane Central Register of Controlled Clinical Trials, CINAHL, and Dissertation Abstracts International), cross-referencing, hand searching, and expert review (Dr. Wendy Kohrt, personal communication). The major key words used in all searches were “exercise,” “bone,” and “randomized.” All searches were conducted by the second author with assistance from a health sciences librarian. The last search was conducted in August, 2011. Detailed search strategies for all databases searched are available upon request from the corresponding author.

### 2.3. Study Selection

 For the current meta-analysis, all studies were selected by both authors. Dual selection of studies was conducted and then reviewed for agreement. Disagreements were resolved by consensus. 

### 2.4. Data Extraction

 Details regarding data extraction procedures have been previously described elsewhere [10–12]. Briefly, codebooks were developed for the extraction of study and participant characteristics. Dual coding was conducted by both authors who reviewed all coded items. Disagreements were resolved by consensus. If consensus could not be reached, the consultant for this project provided a recommendation (Dr. Wendy Kohrt, personal communication). Data from multiple reports on the same participants from the same study was avoided by only including data from the same participants once.

### 2.5. Risk of Bias

Risk of bias was assessed using the Cochrane Risk of Bias assessment instrument [13]. Given the nature of study outcomes, assessment for risk of bias was limited to sequence generation, allocation concealment, and blinding (participants, personnel, and outcome assessors) [13]. Results were categorized as being at a low, high, or unclear risk for bias [13]. Assessments were conducted by both authors with disagreements resolved by consensus. 

### 2.6. Statistical Analysis

#### 2.6.1. Calculation of Effect Sizes from Each Study

The *a priori* primary outcomes for this study were dropouts in the exercise and control groups as well as compliance to the exercise intervention. For all three outcomes, the effect size (ES) of choice was the proportion. Dropouts were defined as the proportion of participants who dropped out of the study after being randomized to the intervention or comparative control group. Compliance was defined as the proportion of required exercise sessions that the exercise participants completed. Each proportion was converted to the logit event rate and its variance prior to pooling. In addition, 95% confidence intervals were generated for each mean event rate for each group from each study.

#### 2.6.2. Effect Size Pooling

All ESs were pooled using a random-effects method of moments model [14]. This approach weights studies by the inverse of the variance and incorporates between-study heterogeneity into the model [14]. All analyses were conducted using the logit event rate and then back transformed to proportions for the purpose of enhancing interpretation and application. Two-tailed 95% confidence intervals (CI) were generated for all findings. Heterogeneity was examined using the *Q* and *I* ^2^ statistics [15, 16]. An alpha value ≤0.10 was considered as statistically significant heterogeneity for *Q* while an *I* ^2^ statistic greater than 50% was considered as excessive inconsistency. 

#### 2.6.3. Sensitivity Analysis

For all three outcomes, multiple groups from the same study were analyzed independently as well as collapsing multiple groups so that only one ES represented each outcome from each study. Outliers, considered to be those with standardized residuals yielding an alpha value ≤0.05, were deleted from the model in order to examine their influence on the overall findings. In addition, influence analysis was conducted with each result deleted from the model once. Small-study effects (publication bias, etc.) were examined using the trim and fill imputation approach (linear estimator *L*) of Duval and Tweedie [17]. In addition, cumulative meta-analysis, ranked by year, was performed in order to examine the accumulation of results over time. 

#### 2.6.4. Moderator Analyses

For categorical variables, mixed-effects, ANOVA-like models for meta-analysis were used to compare potential between-group differences (*Q*_*b*_). A random-effects model was used to combine studies within each subgroup while a fixed-effect model was used to combine subgroups and yield an overall effect. Between-study variance (*τ* ^2^) was not assumed to be equal for all subgroups. For all three outcomes, the *a priori* categorical variables examined included country in which the study was conducted (USA, other), gender, and menopausal status (pre versus post). For exercise dropouts and compliance outcomes, additional moderators examined included type of exercise (aerobic, strength, or both), intensity of exercise (low, moderate, or high), exercise instruction (supervised, unsupervised, or both) and setting (home, facility, or both). Intensity of training for aerobic exercise was classified as low (≤54% of maximal heart rate or ≤39% of either maximum oxygen consumption or maximum heart rate reserve), moderate (55% to 69% of maximal heart rate or 40% to 59% of either maximum oxygen consumption or maximum heart rate reserve), or high (≥70% of maximal heart rate or ≥60% of either maximum oxygen consumption or maximum heart rate reserve). For resistance training, intensity was classified as either low (≤49% of maximal voluntary contraction), moderate (50% to 69% of maximal voluntary contraction), or high (≥70% of maximal voluntary contraction [18, 19]). Potential differences in dropout rates between exercise and control groups were also examined using the mixed-effects model.

For those potential moderators with more than two groups, paired analyses were conducted if the overall between-group difference (*Q*_*b*_) was statistically significant. With the exception of country, potential moderators were selected based on previous research showing an association with dropouts and/or compliance [20–23]. The alpha level for statistical significance was set at *P* ≤ 0.05 while alpha values > 0.05 but ≤0.10 were considered as a trend.

#### 2.6.5. Metaregression

Simple mixed-effects method of moments metaregression was used to examine the association between exercise and control group dropouts as well as compliance to the exercise protocol. Potential predictors included age in years, length of the study in weeks, and the year the study was published. With the exception of publication year, potential moderators were selected based on previous research showing an association with dropouts and/or compliance [20, 24]. The alpha level for statistical significance was set at *P* ≤ 0.05 while alpha values > 0.05 but ≤0.10 were considered as a trend.

#### 2.6.6. Software Used for Statistical Analysis

Data were analyzed using Comprehensive Meta-Analysis (version 2.2) [25], Microsoft Excel 2007 [26], and SSC-Stat (version 2.18) [27].

## 3. Results

### 3.1. Study Characteristics

After screening 1,055 citations, thirty-six studies representing 3,297 participants (1,855 exercise, 1,442 control) were included [28–63]. A flow diagram for the selection of studies is shown in Supplementary File 1 while a general description of the characteristics of each study is shown in Supplementary File 2 (see Supplementary Material available online at http://dx.doi.org/10.1155/2013/250423). Studies were conducted in 11 different countries. These included 14 in the United States [28–60, 60–63], 5 in Canada [33, 34, 48, 56, 61], 4 in Australia [44–46, 55], 3 in Sweden [29, 30, 35], 2 each in the United Kingdom [28, 32], Portugal [50, 51] or Finland [39, 40], and one each in Brazil [31], China [41], Germany [43], and Japan [62].

 Dropout data were available for 51 exercise groups and 39 control groups while compliance to the exercise intervention was available for 31 groups. The number of groups exceeded the number of studies because some studies included more than one exercise and/or control group. The initial number of participants, per group, ranged from 5 to 123 for exercise dropouts ($\overset{-}{X}\pm \text{SD}=36\pm 23$, median = 30), 6 to 123 for control dropouts ($\overset{-}{X}\pm \text{SD}=37\pm 23$, median = 30), and 14 to 123 for exercise compliance groups ($\overset{-}{X}\pm \text{SD}=42\pm 26$, median = 30). 

 Initial characteristics of the participants are shown in Supplementary File 2. The mean between-group age range for all participants was 23 to 83 years ($\overset{-}{X}\pm \text{SD}=56.7\pm 14.7$ years, median = 58.7 years) while initial body weight ranged between 54.1 and 96.2 kg ($\overset{-}{X}\pm \text{SD}=69.7\pm 9.7$ kg, median = 69 kg). Thirty-two of 36 studies (88.9%) were limited to women [28–63], three included both men and women [41, 57, 58], while one was limited to men [46]. For those studies that reported data and included women, 24 of 36 (66.7%) were limited to postmenopausal women [30–35, 37, 38, 41–45, 48, 50–58, 62], 7 to premenopausal women [28, 36, 39, 47, 49, 59, 60], and one to perimenopausal women [29]. For the 20 studies that reported data on race/ethnicity [33, 34, 36, 38, 41–43, 46, 48–54, 57–59, 61, 62], participants, as described by the original study authors, included Whites, Asians, Hispanics, Blacks or African Americans and Indians. While exact numbers could not be elucidated, the majority of participants appeared to be White. With respect to those studies that reported information on cigarette smoking, 13 reported that none of the subjects smoked [34, 35, 37, 39–42, 46, 47, 52, 53, 58, 62] while 11 reported that some did [29, 30, 32, 41, 43, 48, 50–52, 54, 61]. 

A description of the training program characteristics is shown in Supplementary File 2. As can be seen, the exercise interventions varied. Across all intervention groups, length of training ranged from 24 to 104 weeks ($\overset{-}{X}\pm \text{SD}=54.8\pm 24.0$, median = 52) while frequency ranged from 2 to 7 days per week ($\overset{-}{X}\pm \text{SD}=3.8\pm 1.5$, median = 3). For type of exercise, 18 of 51 groups (35%) participated in strength training only, 14 (28%) in aerobic exercise only, and 17 (33%) in both. Another two groups (4%) performed either jumping exercises or a combination of resistance training and agility exercises.

 All of the studies were considered to be at a low risk of bias for sequence generation, an unclear risk for allocation concealment, and a high risk for blinding [28–63].

### 3.2. Outcome Findings

#### 3.2.1. Overall Findings

Overall results for exercise and control dropouts as well as compliance to the exercise intervention are shown in Table 1. As can be seen, exercise group dropouts averaged 21% (95% CI 17% to 26%) with a large amount of heterogeneity. 

**Figure 1:**
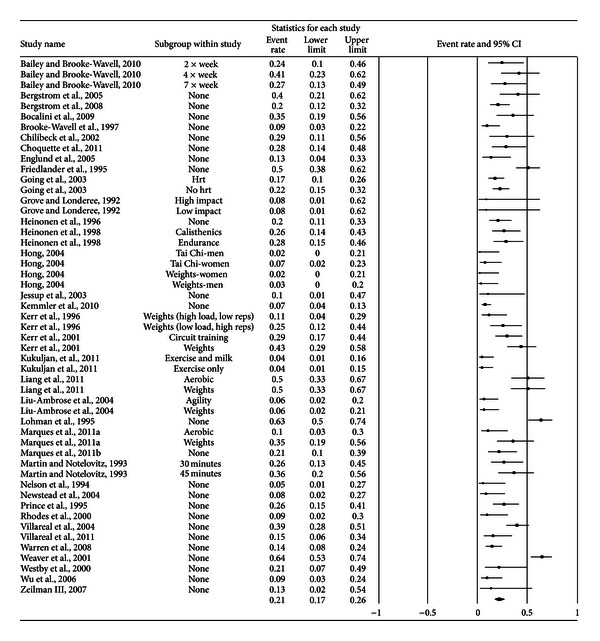
Forest plot for exercise dropouts (group level). Forest plot for exercise dropouts at the group level. The black squares represent the mean event rate while the left and right extremes of the squares represent the corresponding 95% confidence intervals. The middle of the black diamond represents the overall mean event rate while the left and right extremes of the diamond represent the corresponding 95% confidence intervals.

**Figure 2:**
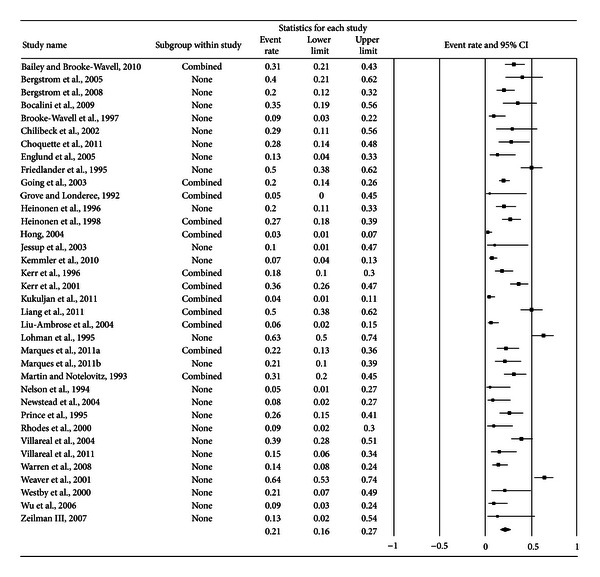
Forest plot for exercise dropouts (study level). Forest plot for exercise dropouts at the study level. The black squares represent the mean event rate while the left and right extremes of the squares represent the corresponding 95% confidence intervals. The middle of the black diamond represents the overall mean event rate while the left and right extremes of the diamond represent the corresponding 95% confidence intervals.

**Figure 3:**
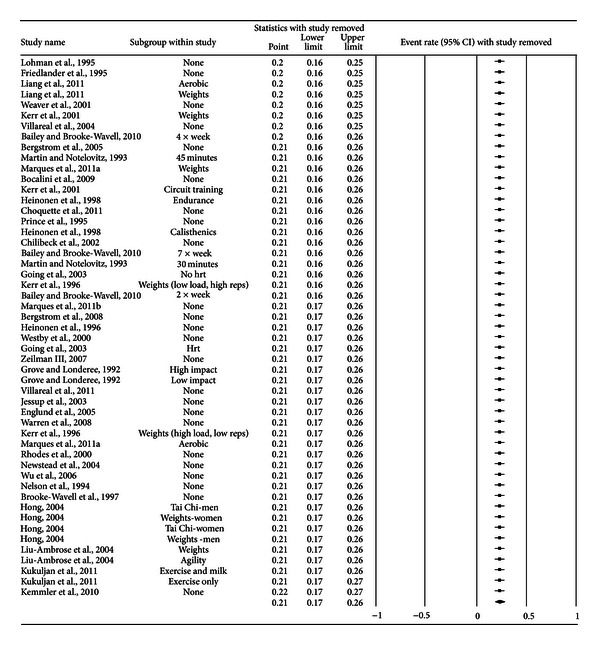
Influence analysis for exercise dropouts. Influence analysis for exercise group dropouts with each group deleted from the model once. The black squares represent the event rate while the left and right extremes of the squares represent the corresponding 95% confidence intervals. The middle of the black diamond represents the overall event rate while the left and right extremes of the diamond represent the corresponding 95% confidence intervals. Results are ordered from the smallest to the largest values.

**Figure 4:**
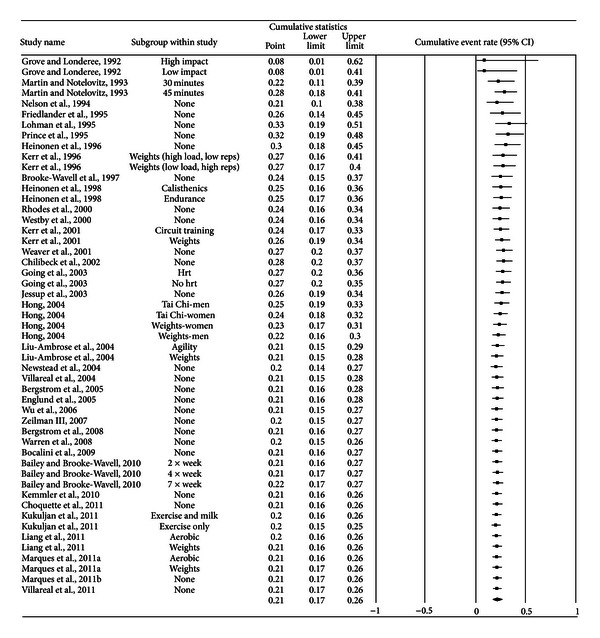
Cumulative meta-analysis for exercise dropouts. Cumulative meta-analysis, ordered by year, for exercise group dropouts. The black squares represent the event rate for each group from each study along with their 95% confidence intervals. The results of each corresponding group are pooled with all studies preceding it. The middle of the black diamond represents the overall mean event rate while the left and right extremes of the diamond represent the corresponding 95% confidence intervals.

A forest plot with the results from each exercise group in each study is shown in Figure 1. Results were similar when findings were collapsed so that only one ES represented each study (Figure 2). With two outlier studies deleted from the model [49, 60], overall results remained similar ($\overset{-}{X}=0.20$, 95% CI, 0.16 to 0.24, *Q* = 163.4, *P* < 0.001, and *I* ^2^ = 70.6%) while heterogeneity was reduced but still large. With each study deleted from the model once, exercise dropouts ranged from only 20% to 21% (Figure 3). No small-study effects (no imputations) were necessary. Cumulative meta-analysis, ranked by year, showed that results have remained consistent for approximately a decade (Figure 4). Reasons for exercise dropouts for the 21 studies that reported such information [28–33, 35, 38–40, 46, 48, 50, 51, 53, 54, 57–59, 62, 63] included time [28, 33, 46, 48, 54], moving [39, 40, 54, 63], loss of interest [39, 40, 50], injuries which may or may not have been related to the exercise intervention [28, 29, 32, 35, 38–40, 54], personal issues [28, 29, 50, 51, 57, 58, 62], medical issues other than injury [29, 32, 46, 48, 50, 51, 53, 57, 58, 62, 63], starting pharmacologic therapy that could affect BMD (hormone replacement therapy, corticosteroids) [29, 59], and pregnancy [39, 59]. Three studies dropped exercise participants because they did not meet their compliance requirements [30, 31, 47].

**Figure 5:**
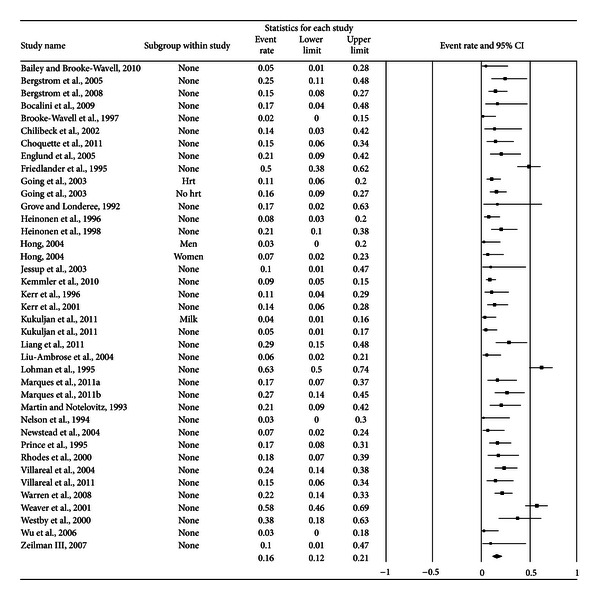
Forest plot for control dropouts (group level). Forest plot for control dropouts at the group level. The black squares represent the mean event rate while the left and right extremes of the squares represent the corresponding 95% confidence intervals. The middle of the black diamond represents the overall mean event rate while the left and right extremes of the diamond represent the corresponding 95% confidence intervals.

**Figure 6:**
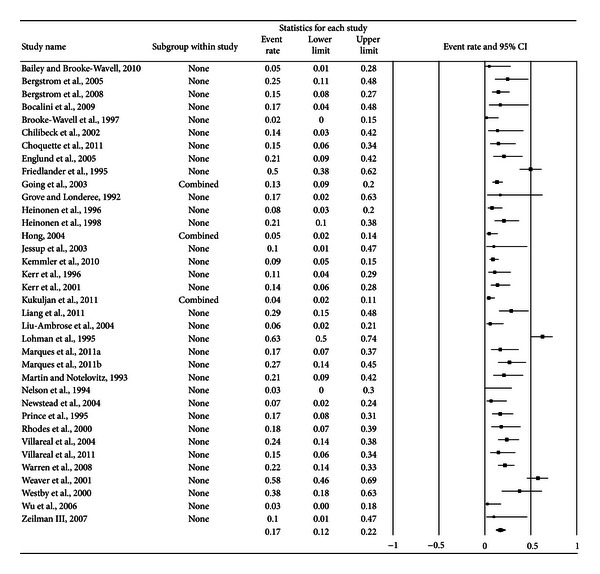
Forest plot for control dropouts (study level). Forest plot for control dropouts at the study level. The black squares represent the mean event rate while the left and right extremes of the squares represent the corresponding 95% confidence intervals. The middle of the black diamond represents the overall mean event rate while the left and right extremes of the diamond represent the corresponding 95% confidence intervals.

**Figure 7:**
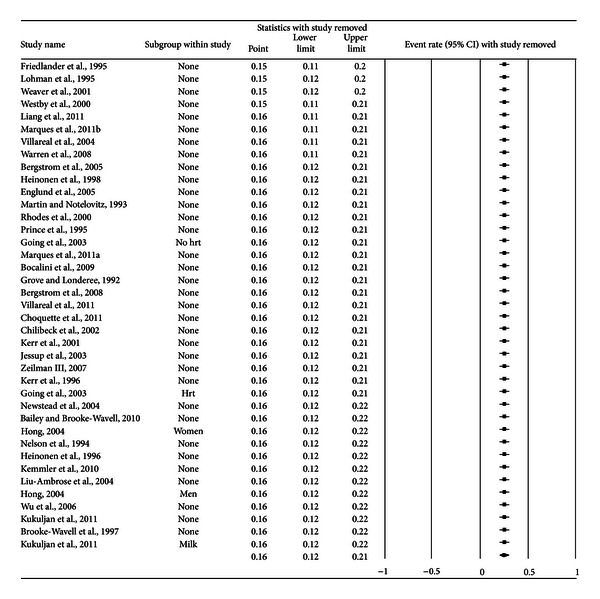
Influence analysis for control dropouts. Influence analysis for control group dropouts with each group deleted from the model once. The black squares represent the event rate while the left and right extremes of the squares represent the corresponding 95% confidence intervals. The middle of the black diamond represents the overall event rate while the left and right extremes of the diamond represent the corresponding 95% confidence intervals. Results are ordered from the smallest to the largest values.

**Figure 8:**
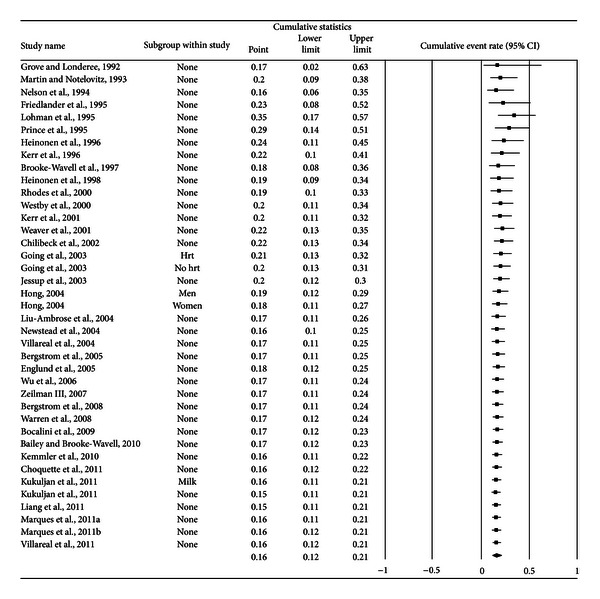
Cumulative meta-analysis for control dropouts. Cumulative meta-analysis, ordered by year, for control group dropouts. The black squares represent the event rate for each group from each study along with their 95% confidence intervals. The results of each corresponding group are pooled with all studies preceding it. The middle of the black diamond represents the overall mean event rate while the left and right extremes of the diamond represent the corresponding 95% confidence intervals.

Control group dropouts averaged 16% (95% CI 12% to 21%). A forest plot with the control group dropout results from each group in each study is shown in Figure 5. Results were similar when findings were collapsed so that only one ES represented each study (Figure 6). With two outlier studies deleted from the model [49, 60], results remained similar to group level findings ($\overset{-}{X}=0.15$, 95% CI 0.12 to 0.19, *Q* = 93.9, *P* < 0.001, and *I* ^2^ = 61.6%). With each study deleted from the model once, the range of control group dropouts was narrow (15% to 16% (Figure 7)). No small-study effects (no imputations) were necessary. Cumulative meta-analysis, ranked by year, showed that results have remained consistent for approximately a decade (Figure 8). No statistically significant differences were observed in dropout rates between exercise and control groups (*Q*_*b*_ = 2.1,  *P* = 0.14). Reasons for control dropouts for the 18 studies that reported such information [28–30, 32, 33, 35, 39, 40, 46, 48, 50, 51, 54, 57–59, 62, 63] included time [46, 48, 54], moving [39, 54], loss of interest [35, 39, 40, 58], personal issues [28, 29, 50, 51, 57], medical issues [32, 33, 39, 50, 51, 57, 58], starting pharmacologic therapy that could affect bone (hormone replacement therapy, corticosteroids) [29, 33, 59], pregnancy [59], started exercising [30, 35], unwilling to serve as a control [50, 51], not available for testing [56], unsatisfied [46], unable to cope with trial [62], and death [35, 40, 57]. 

**Figure 9:**
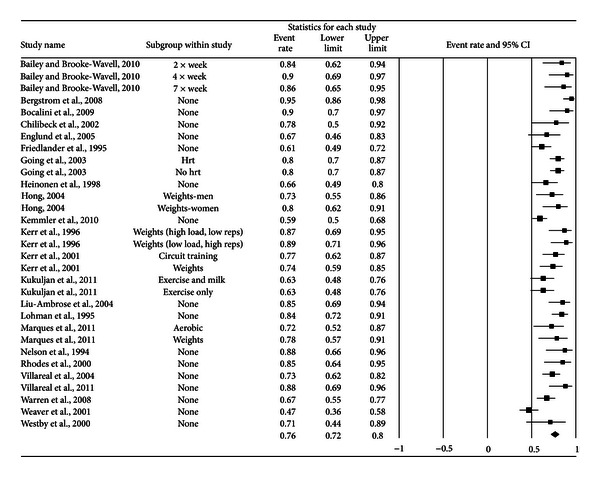
Forest plot for compliance (group level). Forest plot for compliance at the group level. The black squares represent the mean event rate while the left and right extremes of the squares represent the corresponding 95% confidence intervals. The middle of the black diamond represents the overall mean event rate while the left and right extremes of the diamond represent the corresponding 95% confidence intervals.

**Figure 10:**
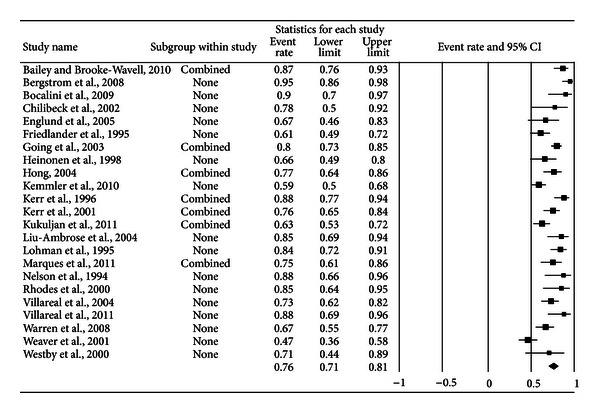
Forest plot for compliance (study level). Forest plot for compliance at the study level. The black squares represent the mean event rate while the left and right extremes of the squares represent the corresponding 95% confidence intervals. The middle of the black diamond represents the overall mean event rate while the left and right extremes of the diamond represent the corresponding 95% confidence intervals.

**Figure 11:**
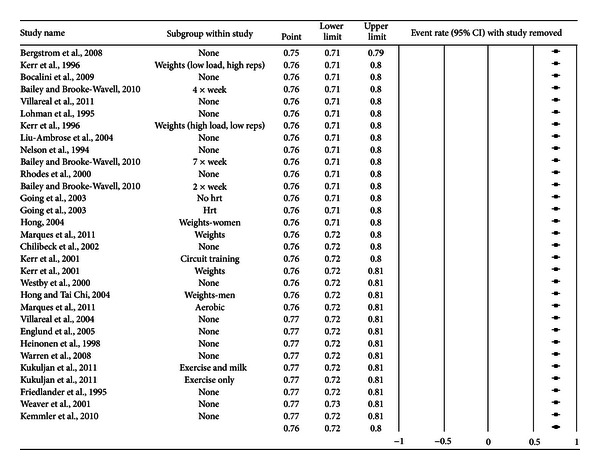
Influence analysis for compliance. Influence analysis for compliance with each group deleted from the model once. The black squares represent the event rate while the left and right extremes of the squares represent the corresponding 95% confidence intervals. The middle of the black diamond represents the overall event rate while the left and right extremes of the diamond represent the corresponding 95% confidence intervals. Results are ordered from the smallest to the largest values.

**Figure 12:**
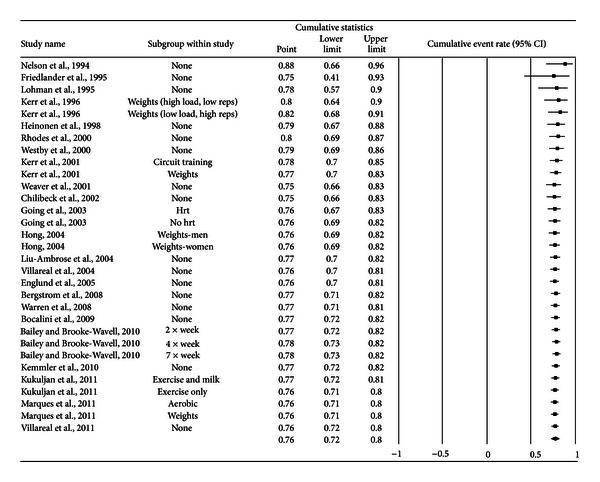
Cumulative meta-analysis for compliance. Cumulative meta-analysis, ordered by year, for compliance. The black squares represent the event rate for each group from each study along with their 95% confidence intervals. The results of each corresponding group are pooled with all studies preceding it. The middle of the black diamond represents the overall mean event rate while the left and right extremes of the diamond represent the corresponding 95% confidence intervals.

Compliance to the exercise intervention averaged 76% (95% CI 72% to 80%). A forest plot with compliance results from each group in each study is shown in Figure 9. Results were similar when findings were collapsed so that only one ES represented each study (Figure 10). With two outlier studies deleted from the model [30, 60], results remained similar to group level findings ($\overset{-}{X}=0.76$, 95% CI 0.72 to 0.80, *Q* = 55.7,  *P* = 0.001, and *I* ^2^ = 49.8%). With each study deleted from the model once, the range for compliance was narrow (75% to 76%) (Figure 11). Adjustment for small-study effects (9 imputations) reduced compliance to 72% (95% CI = 67% to 77%). Cumulative meta-analysis, ranked by year, showed that results have remained consistent for approximately a decade (Figure 12). 

#### 3.2.2. Moderator Analysis

Moderator analyses for dropouts and compliance are shown in Table 2. For both exercise and control groups, dropouts were greater for those studies conducted in the USA versus other countries (exercise = 11%, control = 10%), females versus males (exercise = 19%, control = 12%), and premenopausal versus postmenopausal women (exercise = 22%, control = 18%). There was also a statistically significant difference overall when dropout data for exercise groups were partitioned according to intensity of training while a trend was observed for supervision status. Two-group comparisons demonstrated that dropouts were approximately 13% greater for high- versus moderate-intensity training (*Q*_*b*_ = 7.0,  *P* = 0.008) with no statistically significant differences between high- and low-intensity training (*Q*_*b*_ = 2.3,  *P* = 0.13) or moderate- and low-intensity training (*Q*_*b*_ = 0.48,  *P* = 0.49). For supervision status, dropout rates were approximately 17% greater for unsupervised versus a combination of supervised and unsupervised exercise (*Q*_*b*_ = 5.0,  *P* = 0.03). No statistically significant differences were observed between supervised versus a combination of supervised and unsupervised exercise (*Q*_*b*_ = 1.4,  *P* = 0.24) or supervised and unsupervised exercise (*Q*_*b*_ = 2.4,  *P* = 0.13). No other statistically significant differences were observed for dropouts and any of the other moderators examined. 

 Moderator analysis with respect to exercise compliance demonstrated that adherence to the exercise program was significantly greater (13%) in females versus males. A statistically significant difference was also found for the setting in which exercise took place. Two-group comparisons revealed that compliance was significantly greater (21%) in facility versus a combination of facility- and home-based exercise (*Q*_*b*_ = 4.8,  *P* = 0.03) as well as 28% greater for home versus a combination of facility- and home-based exercise (*Q*_*b*_ = 7.2,  *P* = 0.007). No statistically significant differences were found when data were partitioned according to facility- versus home-based exercise (*Q*_*b*_ = 1.8,  *P* = 0.18). No other statistically significant differences were observed for compliance and any of the other moderators examined.

#### 3.2.3. Regression Analysis

Simple metaregression results for exercise and control group dropouts as well as compliance to the exercise protocol are shown Table 3. For both exercise and control groups, dropout rates were significantly lower with increasing age (*R* ^2^ = 0.32 for exercise and 0.25 for control groups). There was also a statistically significant association between greater dropout rates in the control groups and longer interventions (*R* ^2^ = 0.11). For compliance, longer interventions were significantly associated with lower adherence (*R* ^2^ = 0.31). For all three outcomes, no other statistically significant associations were observed for any of the other potential predictors examined. 

## 4. Discussion

### 4.1. Overall Findings

 The primary purpose of this aggregate data meta-analysis was to examine dropouts and compliance in exercise interventions targeting BMD in adult humans. Collectively, 21% of exercise group participants and 16% of control group participants dropped out of the study while another 24% did not fully comply with the exercise intervention. These results are generally better than pharmacologic interventions aimed at treating osteoporosis. For example, a meta-analysis of 24 large observational studies found that adherence to drug therapies for osteoporosis ranged from 40% to 70% [64]. In addition, one must consider the potential side effects and costs associated with pharmacologic interventions. 

### 4.2. Moderator and Regression Findings

 For both exercise and control groups, dropout rates were significantly greater for studies conducted in the United States versus other countries. While purely speculative, one of the possible reasons for the difference may be related to potentially stricter Institutional Review Board rules in the USA versus other countries, thereby making it easier to withdraw from the study. Alternatively, US participants may be less motivated to maintain a regular exercise program. 

 Greater dropout rates were observed for females versus males in both exercise and control groups while greater compliance was observed for females. These findings suggest that those females who remain in an exercise program are more likely than males to comply with the prescribed program. A recent systematic review by Pavey et al. [20] found that women were more likely than men to begin an exercise referral scheme but less likely to adhere to it. The greater exercise and control group dropout rates found for pre- versus postmenopausal women suggests that older women are less likely to drop out of an exercise program. This is supported by the inverse association that was found between age and dropout rates for both exercise and controls. This finding is further supported by a recent systematic review that found that older people were more likely to begin and adhere to an exercise referral scheme [20]. The lower dropout rate and higher compliance rate for older versus younger participants may reflect a higher level of interest on their part and/or a greater amount of time to devote to exercise. While exercise group dropout rates were progressively greater as the intensity of exercise increased, the only statistically significant difference was between high- and moderate-intensity training. However, the lack of a statistically significant difference between high- and low-intensity training (20% greater for high- versus low-intensity training) may have been the result of the small sample size available (*n* = 3) for low-intensity training. In contrast to the exercise and control group findings, no statistically significant differences were found between compliance and intensity of training. This is in contrast to a recent study that found that the compliance was greater with moderate versus high-intensity aerobic exercise [23]. One of the potential reasons for this difference may be the fact the both aerobic and resistance training interventions were included in the current investigation while the previous study by Perri et al. was limited to one aerobic activity (walking) [23]. Another possible reason for the lack of a statistically significant difference for compliance in the current meta-analysis may have to do with the fact that three included studies dropped exercise participants because they did not meet the study's compliance requirements [30, 31, 47]. It is generally accepted that greater benefits are usually obtained from higher versus lower intensity training programs. However, this needs to be balanced with participant dropout and lower compliance as well as the possibility for an increased risk of injury. Thus, while one should probably not dissuade a participant from higher intensity training, greater adherence may be achieved with moderate (e.g., walking briskly) versus higher (e.g., running) exercise. 

 While there was a trend (*P* = 0.08) for an overall difference in dropout rates between supervised, unsupervised, or combined supervised and unsupervised exercise, subgroup analyses revealed greater dropout rates in unsupervised versus a combination of supervised and unsupervised exercise. However, no statistically significant differences were observed for compliance. This is in contrast to a recent systematic review that suggested that supervised exercise may enhance adherence [21]. While future research in this area appears warranted, it is generally believed that the greater the amount of attention that participants receive, the lower the dropout rate and the greater the compliance. 

 Study length was associated with greater dropout rates in the control groups and poorer compliance in the exercise groups. While no statistically significant association was found for exercise dropouts, results trended in the same direction (*P* = 0.11). The greater dropout rates in the control groups over time may be the result of participants losing interest because of the lack of attention they receive during the study period. It may also be the result of their desire to be assigned to the exercise intervention when they enrolled. The findings of the current meta-analysis support a previous narrative review that found that exercise participation declines over time [24].

 While no statistically significant difference was found between types of exercise (aerobic, strength training, or both), exercise dropouts, and compliance, a recent systematic review with meta-analysis found that resistance exercise predicted higher attendance rates than aerobic exercise in sedentary older adults [22]. The same review also found higher completion rates with facility- versus home-based exercise [22] while the current meta-analysis found greater compliance for facility- or home-based exercise versus both but no difference between home- or facility-based exercise (*P* = 0.18). Based on these findings, it would seem appropriate to suggest that future research in this area is warranted. 

### 4.3. Implications for Research

 The results of the current meta-analysis have several implications for research. For example, the results for dropout and compliance rates may be helpful with respect to sample size estimation for researchers planning future randomized controlled trials. Factors that appear to be important when attempting to minimize dropouts and maximize compliance include (1) gender, (2) age, (3) exercise intensity (low, moderate, or high), (4) exercise supervision (supervised, unsupervised, or both), (5) setting (home, facility, or both), and (6) study length. In addition, researchers can do a better job in reporting the reasons for dropouts. This includes reporting information separately for each group (exercise and control), including the number of participants, per group, associated with the reason for dropping out. Given the lack of information provided by some studies for compliance, future studies should also make sure to report this information. Furthermore, future studies should report the method used to conceal the allocation sequence in sufficient detail to determine whether assignments could have been anticipated prior to or during enrollment. It is also important to note that while blinding was considered to be at a high risk for bias across all studies, it is not possible to blind participants in exercise intervention studies [65]. 

### 4.4. Implications for Practice

The results of the current study suggest that there are several factors that should be considered for practitioners, defined here as those individuals responsible for developing, implementing, and evaluating exercise programs. These include (1) gender, (2) age, (3) exercise intensity (low, moderate, or high), (4) exercise supervision (supervised, unsupervised, or both), and (5) setting (home, facility, both). While maximizing long-term participation is important, this has to be balanced with available resources. 

### 4.5. Limitations

 While the results of the current meta-analysis provide some interesting findings, they should be interpreted with respect to the following potential limitations. First, because of missing data for different variables from different studies as well as small sample sizes within selected subgroups, multiple meta-regression analysis was not performed. As a result, potential confounding factors were not controlled for. Second, one or more of the statistically significant findings may have been nothing more than the play of chance given the large number of statistical tests conducted. However, an *a priori* decision was made to not adjust alpha values because such adjustments tend to be overly conservative [66]. Furthermore, the investigative team did not want to miss any potentially important findings that might be worthy of further investigation [66]. Third, while the current study included a number of potential moderators, many other potential determinants of exercise adherence exist but are not typically reported in randomized controlled trials. Broadly, these include such things as personal (self-efficacy, enjoyment of activity, etc.) and environmental (spousal support, disruptions in routine, etc.) characteristics [24]. Finally, the results of the current meta-analysis may not be generalizable beyond the participants and interventions that were included. 

## 5. Conclusions

 The current study addresses a recent recommendation for additional research on the determinants of exercise [5]. It provides important evidence regarding dropouts and compliance, including factors associated with dropouts and compliance, for exercise interventions targeting BMD in adult humans. However, additional randomized controlled trials that focus on dropouts and compliance are needed. 

## Supplementary Material

Supplementary file 1 describes the flow of information through the review process, including the reasons for those studies that were excluded. A reference list of excluded studies is available on request from the corresponding author
Supplementary file 2 provides a general description of the characteristics of the included studies. A reference list of excluded studies is available on request from the corresponding author.

## Figures and Tables

**Table 1:** Overall dropout and compliance results.

Variable^a^	Studies	No. ES/P	$\overset{-}{X}$ (95% CI)	*Q*(*p*)	*I* ^2^ (%)
(i) Exercise dropout	36	51/1855	0.21 (0.17, 0.26)	242.6 (<0.001)*	79.4
(ii) Control dropout	36	39/1442	0.16 (0.12, 0.21)	187.2 (<0.001)*	79.7
(iii) Compliance	23	31/1317	0.76 (0.72, 0.80)	90.0 (<0.001)*	66.7

^a^Outcomes reported as proportions; No. ES/P: number of effect sizes and participants nested within ESs; Q(*p*): Cochran's *Q* statistic and alpha value; *I* ^2^ (%): *I*-squared; *statistically significant (*P*≤ 0.05).

**Table 2:** Moderator analyses (mixed effects) for dropouts and compliance.

Variable	Dropouts (exercise)			Dropouts (control)			Compliance		
	No. ES/P	$\overset{-}{X}$ (95% CI)	*Q*_*b*_(*p*)	No. ES/P	$\overset{-}{X}$ (95% CI)	*Q*_*b*_(*p*)	No. ES/P	$\overset{-}{X}$ (95% CI)	*Q*_*b*_(*p*)
Country									
USA	18/732	0.29 (0.20, 0.39)	4.5 (0.03)*	15/606	0.23 (0.15, 0.35)	4.5 (0.03)*	9/565	0.74 (0.65, 0.82)	0.40** **(0.53)
Other	33/1123	0.18 (0.14, 0.23)		24/836	0.13 (0.10, 0.17)		22/752	0.77 (0.72, 0.82)	
Gender									
Females	44/1601	0.23 (0.18, 0.28)	18.1 (<0.0001)*	33/1236	0.17 (0.13, 0.23)	9.3 (0.002)*	26/1101	0.78 (0.72, 0.82)	6.4 (0.01)*
Males	5/159	0.04 (0.02, 0.09)		4/129	0.05 (0.02, 0.10)		3/121	0.65 (0.56, 0.73)	
Menopausal status									
Premenopausal	10/448	0.40 (0.28, 0.53)	12.1 (0.001)*	7/358	0.32 (0.18, 0.50)	6.5 (0.01)*	7/337	0.74 (0.61, 0.84)	0.92** **(0.34)
Postmenopausal	32/1147	0.18 (0.14, 0.23)		25/885	0.14 (0.12, 0.17)		19/810	0.80 (0.75, 0.84)	
Type of exercise									
Aerobic	14/386	0.19 (0.13, 0.27)		NA	NA		1/24	0.72 (0.52, 0.87)	
Strength	18/556	0.24 (0.16, 0.34)	0.77 (0.68)	NA	NA	NA	17/526	0.80 (0.75, 0.83)	4.2** **(0.12)
Both	17/852	0.22 (0.15, 0.31)		NA	NA		13/767	0.71 (0.64, 0.78)	
Exercise intensity									
Low	3/88	0.07 (0.01, 37)		NA	NA	NA	3/88	0.80 (0.69, 0.88)	
Moderate	11/354	0.14 (0.08, 0.21)	8.6 (0.01)*	NA	NA		7/225	0.72 (0.64, 0.80)	1.4** **(0.50)
High	25/1071	0.27 (0.21, 0.35)		NA	NA	NA	19/902	0.76 (0.69, 0.81)	
Exercise supervision									
Supervised	34/1280	0.21 (0.16, 0.27)		NA	NA	NA	22/972	0.76 (0.71, 0.80)	
Unsupervised	8/244	0.32 (0.19, 0.48)	5.1 (0.08)**	NA	NA		6/216	0.82 (0.59, 0.93)	1.7** **(0.42)
Both	9/331	0.15 (0.10, 0.23)					3/129	0.70 (0.59, 0.79)	
Setting									
Home	5/87	0.28 (0.20, 0.39)		NA	NA		4/79	0.83 (0.73, 0.90)	
Facility	35/1350	0.20 (0.15, 0.26)	3.0 (0.22)	NA	NA	NA	23/104	0.76 (0.71, 0.80)	7.2 (0.02)*
Both	7/271	0.15 (0.05, 36)		NA	NA		22/101	0.55 (0.35, 0.73)	

Data reported as proportions; No. ES/P: number of effect sizes and participants nested within ESs; $\overset{-}{X}$ (95% CI): mean and 95% confidence interval; *Q*_*b*_(*p*): between-group difference (*Q*_*b*_) and alpha value (*p*); NA: not applicable; *statistically significant at *P*≤ 0.05; **trend for statistical significance (*P* > 0.05 to ≤0.10).

**Table 3:** Meta regression results for dropouts and compliance.

Variable	No. ES/P	*β*_1_± SE	*Z*(*p*)
Dropouts (exercise)			**—**
Age (years)	51/1855	−0.003 ± 0.007	−4.7 (<0.001)*
Study Length (wks)	51/1855	0.009 ± 0.005	1.6 (0.11)
Year published	51/1855	−0.02 ± 0.02	−0.86 (0.39)
Dropouts (control)			
Age (years)	39/1442	−0.03 ± 0.009	−3.5 (0.0005)*
Study length (wks)	39/1442	0.01 ± 0.007	2.0 (0.05)*
Year published	39/1442	−0.04 ± 0.03	−1.5 (0.12)
Compliance			
Age (years)	31/1317	0.007 ± 0.007	.96 (0.34)
Study length (wks)	31/1317	−0.01 ± 0.004	−3.5 (0.0004)*
Year published	31/1317	−0.004 ± 0.02	−.21 (0.83)

No. ES/P: number of effect sizes and participants nested within ESs; *β*_1_± SE: slope ± standard error; *Z*(*p*): *z-*score and alpha value; *statistically significant (*P*≤ 0.05); **trend for statistical significance (*P*>.05 to ≤.10).
