# Supplementary figures and images for: Dropouts and Compliance in Exercise Interventions Targeting Bone Mineral Density in Adults: A Meta-Analysis of Randomized Controlled Trials
Source: J Osteoporos. 2013 Jun 3;2013:250423. doi: 10.1155/2013/250423 (PMC3686150; doi:10.1155/2013/250423)

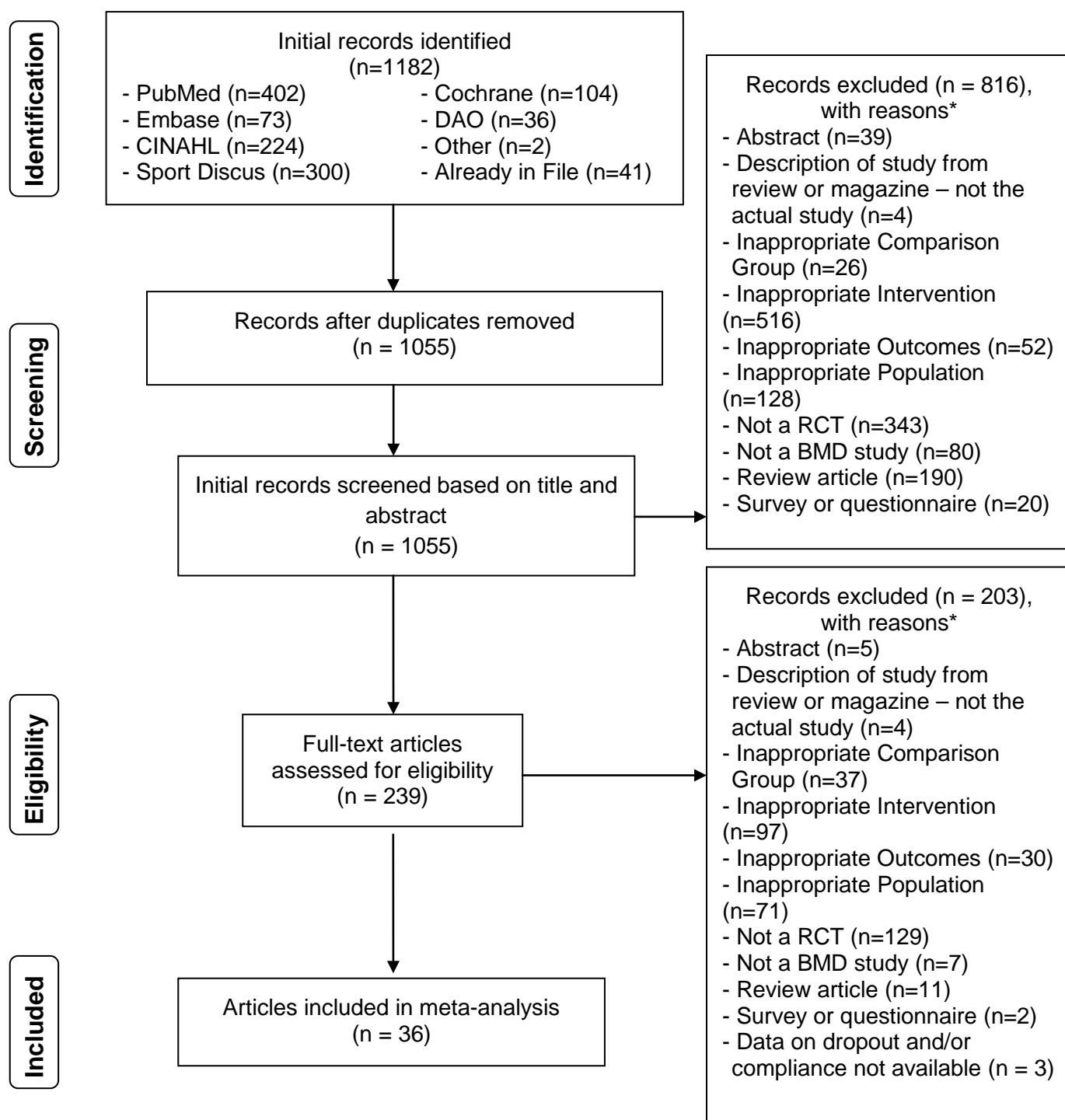

Supplementary File 1. Flow diagram for the selection of studies.

Supplement: Supplementary file 1 — describes the flow of information through the review process, including the reasons for those studies that were excluded. A reference list of excluded studies is available on request from the corresponding author Supplementary file 2 provides a general description of the characteristics of the included studies. A reference list of excluded studies is available on request from the corresponding author. [file 250423.f1.pdf]
