# Supplementary material for: Dropouts and Compliance in Exercise Interventions Targeting Bone Mineral Density in Adults: A Meta-Analysis of Randomized Controlled Trials
Source: J Osteoporos. 2013 Jun 3;2013:250423. doi: 10.1155/2013/250423 (PMC3686150; doi:10.1155/2013/250423)
Supplement: Supplementary file 2 [file 250423.f2.pdf]

Supplementary File 2. General Characteristics of Studies.

Study	Country	Participants	Intervention
Bailey and Brooke-Wavell, 2010[28]	United Kingdom	85 healthy, premenopausal women 18 to 45 yrs of age assigned to 0 (n = 20), 2 (n = 21), 4 (n = 22) or 7 (n = 22) days/wk of exercise	2, 4, or 7 days/wk of 5 sets of 10 hops on one limb with 15 seconds of walking between each set for 6 months
Bergstrom et al., 2005[29]	Sweden	40 perimenopausal women assigned to a physical training (n=20, 44-51 yrs of age),or control (n=20, 41-51 yrs of age) group	3 fast 30-min walks plus 2 sessions of 1h training (5 min warm-up, 25 min strengthening exercise for arms, legs, back & stomach, 25 min aerobic exercise, 5 min stretching) per wk for 18 months
Bergstrom et al., 2008[30]	Sweden	112 postmenopausal women 45 to 65 yrs of age assigned to either a physical training (n=60) or control (n=52) group	3 fast 30-min walks and 1 to 2 sessions of 1h training (5 min warm-up, 25 min strengthening exercise for arms, legs, back & stomach, 25 min aerobic exercise, 5 min stretching) per wk for 1 yr
Bocalini et al., 2009[31]	Brazil	35 postmenopausal women assigned to either a trained (n=23, age, $\bar{X}$ ± SD= 69 $\pm$ 34.86 yrs) or an untrained control (n=12, age, $\bar{X}$ ± SD= 67 $\pm$ 25.29 yrs) group	3 resistive exercise sessions/wk, 1 h/session, 12 exercises, 3 sets of 10 reps at 85% 1RM, for 24 wks
Brooke-Wavell et al.,1997[32]	United Kingdom	84 healthy, postmenopausal women 60 to 70 yrs of age assigned to either a walking (n=43) or control (n=41) group	Brisk walking for 280 min/wk, each session 20-50 min, $\bar{X}$ ± SD= 20.4 $\pm$ 3.8 min/day, for 1 yr

Chilibeck et al., 2002[33]	Canada	28 postmenopausal women assigned to either an exercise + placebo (n=14, $\bar{X} \pm$ SD= 56.8 $\pm$ 2 yrs of age) or placebo (n=14, $\bar{X} \pm$ SD= 58.8 $\pm$ 1.8 yrs of age) group	Strength training, 3 days/wk, 12 exercises, 2 sets, 8-10 reps, 70% 1RM, for 1 yr
Choquette et al., 2011[34]	Canada	51 healthy overweight or obese, postmenopausal women 50 to 70 yrs of age assigned to either an exercise + placebo (n=25) or placebo (n=26) group	3 days/wk, 1 h/session: 30 min. aerobic exercise (70-85% HRR on cycle or treadmill) plus 30 min resistance training (4 sets, 4-6 reps, 60-85% 1RM), for 6 months
Englund et al., 2005[35]	Sweden	48 postmenopausal women 66 to 87 yrs of age assigned to either an exercise (n=24) or control (n=24) group	2 days/wk, 50 min/session: aerobic walk, jog or stepping (10 min), strengthening (2 sets, 8-12 reps for each muscle group, 12 min), dynamic & static balance & coordination exercises (5 min) plus warm-up & cool down, for 47 wks
Friedlander et al., 1995[36]	United States	126 women 20 to 35 yrs of age assigned to either an exercise (n=64) or stretching (n=62) group	3 days/wk, 1 h/session, alternating classes of circuit training, strength training, and aerobic exercise (70-85% of VO_{2max}), for 2 yrs
Going et al., 2003[37]	United States	320 healthy, postmenopausal women 40 to 65 yrs of age assigned to an exercise + HRT (n=86), exercise + no HRT (n=91), control + HRT (n=73), or control + no HRT (n=70) group	3 days/wk, Weightlifting (7 exercises, 2 sets, 6-8 reps, 70-80% 1RM), Aerobic weight-bearing activity circuit (20-25 min of walk, jog, skip, hop, & stair climb/step box with weighted vest) at 60% of MHR, for 1 yr
Grove &	United	16 healthy, sedentary, early postmenopausal	3 days/wk, 1 h/session (15-20 min warm-up, 20 min of either low

Londeree, 1992[38]	States	women 49 to 64 yrs of age assigned to a low impact exercise (n=5), high impact exercise (n=5) or control (n=6) group	impact or high impact activities, 15 min cool down), for 1 yr
Heinonen et al.,1996[39]	Finland	98 healthy, sedentary premenopausal women 35 to 40 yrs of age assigned to either a training (n=49) or control (n=49) group	3 days/wk, 1 h/session (15 min warm-up, 20 min high impact jump training, 15 min calisthenics, 10 min cool down), for 18 months
Heinonen et al., 1998[40]	Finland	101 healthy, sedentary, perimenopausal women 52 to 53 yrs of age assigned to an endurance (n=32), calisthenics (n=35) or control/stretching (n=34) group	Endurance: 3.2 days/wk, 50 min/session, walk, jog, cycle, or stair climb at 55-75% max VO_2; Calisthenics: 2.6 days/wk, 50 min/session, 3 sets of 16 reps of 8 rhythmic muscular strength-endurance exercises, for 18 months
Hong, 2004 Hong [41]	China	180 healthy men & women 65 to 74 yrs of age assigned to a Tai Chi (men, n=30; women, n=30), resistance training (men, n=30; women, n=30) or control (men, n=30; women, n=30) group	3 days/wk: Tai Chi: Yang style, 24 forms, 45 min; Resistance Training: 1 set, 30 reps, 7 exercises, therabands used for resistance; for 12 months
Jessup et al., 2003[42]	United States	20 women, $\overline{X} \pm$ SD, 69.2 $\pm$ 3.5 yrs of age assigned to either an exercise (n=10) or control (n=10) group	3 days/wk, 60-90 min/session, Strength Training (8-10 reps at 50-75% 1RM) and 30-45 min walking, stair climbing, & balance exercises wearing weighted vests, for 32 weeks
Kemmler et al., 2010[43]	Germany	246 women assigned to either an exercise (n=123, $\overline{X} \pm$ SD= 68.9 $\pm$ 3.9 yrs of age) or	2, 60-min supervised classes (20 min aerobic dance at 70-85% max $VO_{2;}$ 5 min balance training; functional gymnastics/isometric

		placebo (n=123, $\bar{X}$ ± SD= 69.2 ± 4.1 yrs of age) group	training/stretching: 1-3 sets, 10-15 reps, 6 exercises; 2-3 sets of 3 upper body exercises (10-15 reps, 9 exercises) and 2 40-min home sessions (1-2 sets, 10-15 reps, 8-11 exercises), for 18 months
Kerr et al., 1996[44]	Australia	56 postmenopausal women assigned to either an endurance strength (n=28, $\bar{X}$ ± SD= 55.7 ± 4.7 yrs of age) or a strength (n=28, $\bar{X}$ ± SD=58.4 ± 3.7 yrs of age) group (one side of body was exercised; the other side was control)	3 days/wk, 3 sets,12 exercises; Endurance: 45-60 min/session, 8 reps, 60% 1RM for leg, 40% 1RM for arm; Strength: 20-30 min/session, 20 reps, 20% 1RM for leg, 10% 1RM for arm; for 1 yr
Kerr et al., 2001[45]	Australia	126 postmenopausal women $\bar{X}$ ± SD= 60 ± 5 yrs of age assigned to a strength (n=42), fitness (n=42) or control (n=42) group	3 days/wk, 1 h/session, 3 sets, 9 exercises; Strength: 8 reps, Fitness: 40 seconds per station plus 40 seconds stationary cycling, for 2 yrs
Kukuljan et al., 2011[46]	Australia	180 healthy men 50 to 79 yrs of age assigned to either an exercise only (n=46), exercise + milk (n = 45), control (n=44) or control and milk (n = 45) group	3 days/wk, 60-75 min/session, Resistance Training: 2-3 sets, 8-20 reps, 50-85% 1RM, 6-8 exercises plus 3 moderate-impact weight-bearing exercises (jumping & stepping) in between resistance exercises 3 sets of 10-20 reps, for 18 months
Liang et al., 2011[47]	United States	90 healthy, untrained women 20 to 35 yrs of age assigned to a strength training (n=30), step aerobics (n=32), or control (n=28) group	3 days/wk, 40 min/session, Strength: 1-3 sets, 8-15 reps, 65-80% 1RM, 8 exercises; Step Aerobics: step, hop, walk, run in place, 20 cm step height, 15-300 hop cycles/session, for 12 months
Liu-Ambrose et	Canada	104 women 75 to 85 yrs of age assigned to a	2 days/wk, 50-min/session, Resistance: 2 sets of 10-15 reps at

al., 2004[48]		resistance training (n=34), agility training (n=36) or stretching (control) (n=34) group	50-85% 1RM, 9 exercises; Agility: improve balance, coordination & reaction time via ball games, relays, dance & obstacle courses, for 25 wks
Lohman et al., 1995[49]	United States	118 premenopausal women 28 to 39 yrs of age assigned to either an exercise (n=59) or control (n=59) group	3 days/wk, 1 h/session, 3 sets, 8-12 reps, 70-80% 1RM, 12 weight lifting exercises, 18 months
Marques et al., 2011[50]	Portugal	71 women 61 to 83 yrs of age assigned to a resistance (n=23), aerobic (n=24) or control (n=24) group	3 days/wk, 60 min/session, Aerobic: 50-85% of HRR for 35-40 min of each session: step, skip, walk, jog, dance, aerobic, step; Resistance: 2 sets, 6-15 reps, 50-85% 1RM, 8 exercises, for 32 wks
Marques et al., 2011[51]	Portugal	60 women assigned to either an exercise (n=30, $\bar{X}$ ± SD=70.1 ± 5.4 yrs of age) or control (n=30, $\bar{X}$ ± SD=68.2 ± 5.7 yrs of age) group	2 days/wk, 60 min/session, consisting of 15 min weight-bearing activities, 10 min muscular endurance (3 sets, 8-15 reps), balance & agility training, for 32 wks
Martin & Notelovitz, 1993[52]	United States	76 postmenopausal women assigned to a 30 min exercise (n=27, $\bar{X}$ ± SD=60.3 ± 7.8 yrs of age), 45 min exercise (n=25, $\bar{X}$ ± SD=57.8 ± 7.1 yrs of age) or control (n=24, $\bar{X}$ ± SD=56.7 ± 6.9 yrs of age) group	Aerobic exercise, 3 days/wk, 30 or 45 min/session, 70-85% MHR on treadmills, for 12 months
Nelson et al., 1994[53]	United States	40 sedentary, postmenopausal women 50 to 70 yrs of age assigned to either an exercise (n=21)	2 days/wk of strength training, 45 min/session, 3 sets, 8 reps, 80% 1RM, 5 exercises, for 1 yr

		or control (n=19) group	
Newstead et al., 2004[54]	United States	53 postmenopausal women 50 to 65 yrs of age assigned to either an exercise (n=25) or control (n=28) group	3 jumping sessions/wk on the floor and from aerobic steps (4 and 6 inches), 25-200 jumps/session, for 1 yr
Prince et al., 1995[55]	Australia	84 postmenopausal women 50 to 70 yrs of age assigned to either a calcium + exercise (n=42) or calcium only (n=42) group	4 h/wk of weight-bearing exercise (2 h classes, 2 h walking on own) at 60% of MHR, for 2 yrs
Rhodes et al., 2000[56]	Canada	44 healthy, sedentary women 65 to 75 yrs of age assigned to either an exercise (n=22) or control (n=22) group	Resistance training, 3 days/wk, 1 h/session, 3 sets, 8 reps, 75% 1RM, 6 exercises, for 1 yr
Villareal et al., 2004[57]	United States	119 sedentary men & women 78 yrs of age or older assigned to either an exercise (n=69) or home training (n=50) group	2.2 days/wk for 9 months; Phase 1: physical therapy, flexibility, coordination & balance. Phase 2: Resistance: 1-3 sets, 6-12 reps, 65-100% 1RM, 8 exercises; Phase 3: endurance: walk, cycle, row, 15-30 min/session, 65-90% MHR; HOME: 2.9 days/wk flexibility exercises
Villareal et al., 2011[58]	United States	53 obese men & women 65 yrs of age or older assigned to either an exercise (n=26) or control (n=27) group	3 days/wk, 90 min/session, Aerobic Exercise (walk, cycle, stair climb) 65-85% MHR, Resistance Training (1-3 sets, 6-12 reps, 65-80% 1RM, 9 exercises), flexibility & balance exercises, for 1 yr

Warren et al., 2008[59]	United States	148 healthy, sedentary, overweight premenopausal women 25 to 44 yrs of age assigned to either an exercise (n=72) or control (n=76) group	2 days/wk, strength training, 3 sets, 8-10 reps, for 2 yrs
Weaver et al., 2001[60]	United States	141 women 18 to 31 yrs of age assigned to either an exercise (n=77) or control (n=64) group	3 days/wk of super circuit resistance training, 8 upper and 8 lower body exercises with a cycle ergometer between each station, 8-12 reps, 70% 1RM, plus 60 min of jumping rope/wk, for 24 months
Westby et al., 2000[61]	Canada	30 women with rheumatoid arthritis taking low-dose prednisone assigned to either an exercise (n=14, $\overline{X}$ ± SD=56.4 $\pm$ 10.1 yrs of age) or control (n=16, $\overline{X}$ ± SD=56 $\pm$ 10.8 yrs of age) group	Aerobic dance & strengthening program, 2.1 days/wk, 45-60 min/session, 60-75% MHR, for 1 yr
Wu et al., 2006[62]	Japan	68 healthy, postmenopausal women 45 to 60 yrs of age assigned to either a placebo + walking (n=34) or placebo only (n=34) group	3 days/wk, 45-min/session, walking at a speed of 5-6 km/hr, monitored by a pedometer, for 24 wks
Zeilman, 2007[63]	United States	18 sedentary men with irritable bowel syndrome ages 21 to 75 yrs of age assigned to either an exercise (n=8) or control (n=10) group	3 days/wk, 50 min/session, stretching, flexibility calisthenics & walking with weighted vests and a pedometer, for 32 wks

Notes: yrs, years; min, minute(s); h, hour(s); wks, weeks; wk, week; km, kilometer; RM, repetition maximum; reps, repetitions; VO_{2max}, maximum oxygen consumption; MHR, maximum heart rate; HRR, heart rate reserve; HRT, hormone replacement therapy; Description of groups limited to those that met the inclusion criteria for the current meta-analysis.
